# Atad1 Is a Potential Candidate Gene for Prepulse Inhibition

**DOI:** 10.3390/genes16101139

**Published:** 2025-09-26

**Authors:** Akhilesh K. Bajpai, Timothy G. Freels, Lu Lu, Melloni N. Cook

**Affiliations:** 1 Department of Genetics, Genomics and Informatics, University of Tennessee Health Sciences Center, Memphis, TN 38163, USA; abajpai3@uthsc.edu; 2 Department of Psychology, The University of Memphis, Memphis, TN 38152, USA; timothy.freels@umassmed.edu

**Keywords:** prepulse inhibition, Atad1, candidate gene, schizophrenia, BXD RI strains

## Abstract

**Background/Objectives**: Prepulse inhibition (PPI) is a robust, reproducible phenotype associated with schizophrenia and other psychiatric disorders. This study was carried out to identify gene(s) influencing PPI. **Methods**: We performed Quantitative Trait Locus (QTL) analysis of PPI in 59 strains from the BXD recombinant inbred (BXD RI) mouse family and used a 2-LOD region for candidate gene identification. Genes significantly correlated with the candidate gene were identified based on genetic, partial, and literature correlation, and were further studied through gene enrichment and protein–protein interaction analyses. Phenome-wide association study (PheWAS) and differential expression analyses of the candidate gene were performed using human data. **Results**: We identified one significant (GN Trait 11428) and two suggestive male-specific QTLs (GN Traits 11426 and 11427) on Chromosome 19 between 27 and 36 Mb with peak LRS values of 19.2 (−logP = 4.2), 14.4 (−logP = 3.1), and 13.3 (−logP = 2.9), respectively. *Atad1*, ATPase family, AAA domain containing 1 was identified as the strongest candidate for the male-specific PPI loci. *Atad1* expression in BXDs is strongly *cis*-modulated in the nucleus accumbens (NAc, LRS = 26.5 (−logP = 5.7). Many of the *Atad1*-correlated genes in the NAc were enriched in neurotransmission-related categories. Protein–protein interaction analysis suggested that ATAD1 functions through its direct partners, GRIA2 and ASNA1. PheWAS revealed significant associations between *Atad1* and psychiatric traits, including schizophrenia. Analysis of a human RNA-seq dataset revealed differential expression of *Atad1* between schizophrenia patients and the control group. **Conclusions**: Collectively, our analyses support *Atad1* as a potential candidate gene for PPI and suggest that this gene should be further investigated for its involvement in psychiatric disorders.

## 1. Introduction

Schizophrenia affects approximately 1.1% of the U.S. adult population, with associated lifetime prevalence estimates ranging from 0.3 to 1% [1,2,3], and is characterized by numerous behavioral and brain abnormalities (e.g., impaired social cognition, delusions, flattened affect, and region-specific changes in brain volume, etc.). Although widely studied, the etiology of schizophrenia remains poorly understood. One of the first large-scale GWAS on schizophrenia identified 108 significant schizophrenia-related loci [4,5]; after increasing the size of the cohort, the number of significant loci increased to 270 [6]. Schizophrenia is highly heritable, with estimates between 79 and 87% [7,8,9]. Prepulse inhibition (PPI), an index of sensory motor gating, has been advanced as a reliable endophenotype for schizophrenia-related behavior [10] and is moderately heritable, with estimates ranging from 31 to 50% [11,12,13,14,15]. Interestingly, deficits in PPI are detectable prior to the onset of psychosis [13], suggesting usefulness in assessing risk for development of psychosis. A recent study reported decreased neural PPI in early schizophrenia and bipolar disorder patients [16]. Identifying the genetic components contributing to psychosis may aid in our understanding of schizophrenia and in the development of therapeutic agents. Critically, identification of biomarkers associated with schizophrenia risk may permit earlier detection/diagnosis.

Mouse models have been useful in identifying genetic factors contributing to schizophrenia. *Disc1* (disrupted in schizophrenia 1) and *Nrg1* (Neuregulin 1) are among the genes most often associated with schizophrenia in mouse studies, as reviewed in [17]. Still, many models are of single gene mutations and do not address interactions between multiple genes. There are likely many genes that contribute with a small effect to schizophrenia. One way of identifying these genes is examination of schizophrenia-related endophenotypes. PPI and sensorimotor gating phenotypes have been associated with schizophrenia-related behavior, other mental disorders [18], Alzheimer’s disease [19], anxiety, Huntington’s disease, and autism among others, as reviewed in [20]. Thus, additional insight into genetic regulation of PPI/sensorimotor gating could prove informative with respect to multiple disorders.

Acoustic startle and PPI tasks assess startle response and sensorimotor gating. The startle reflex is measured in response to a loud acoustic stimulus, while a prepulse stimulus preceding the acoustic stimulus should inhibit a startle response to the acoustic stimulus [21,22]. There are several lines of evidence showing that PPI is genetically modulated. Studies have identified PPI QTLs in models including the chromosome (Chr) substitution strains Chr. 16, Ref. [23], and Chrs 4, 10, 11, and 16, Ref. [24]; the recombinant congenic strains Chrs 3, 5, 7, and 16, Ref. [25]; the heterogeneous stock Chrs 11 and 16, Ref. [26]; F_1_- and F_2_-crosses and advanced intercross lines Chr 7, Ref. [27], Chrs 2 and 7, Ref. [28], and Chrs 11 and 12 [29]; and a limited number of BXD recombinant inbred (RI) strains, including Chr 17, Ref. [30]. The power to dissect genetic regulation of traits like PPI/sensorimotor gating often lies in the model(s) used. Here, we used a powerful genetic resource, BXD RI strains, to examine genetic regulation of PPI.

The original BXD RI panel consisted of approximately 30 extant strains, providing advantages over smaller sets of RI strains, but still limiting precision and power to detect QTLs of small effect size [31]. After the addition of advanced intercross RI strains to this panel [32], as well as more recent additions, the expanded BXD RI panel is now the largest mouse RI panel. The increased number of strains offers significant promise in mapping QTLs of large and small effect sizes, influencing virtually any phenotype of interest. This expanded panel also allows for analysis of epistatic interactions, something not easily accomplished with the original set [32]. The BXD RI strains have been genotyped with more than five million microsatellite and single-nucleotide polymorphic (SNP) markers; thus, there is a dense genetic map of this panel. Importantly, the advanced intercross RI strains have more recombination events, permitting greater mapping precision.

## 2. Materials and Methods

### 2.1. Animals

Male and female mice from 59 BXD RI strains were used for behavioral testing. A total of 455 animals (2–6 animals per sex per strain) were tested. All animals were 45–55 days of age at the time of testing. The study was approved by the University of Memphis Institutional Animal Care and Use Committee. Additional details on breeding and housing are provided in [33].

### 2.2. Acoustic Startle and Prepulse Inhibition

We carried out behavioral testing as previously described [33,34]. Briefly, 55 pseudorandom trials consisted of an acoustic startle stimulus (120 dB white noise burst) and prepulse stimuli (70-, 80-, and 85 dB white noise bursts, 20 msec in duration) which preceded the startle stimulus by 100 msec. All data (means and standard errors) generated by us were submitted to GeneNetwork (www.genenetwork.org) and are publicly available. This dataset was revisited after more advanced genetic, sequencing and bioinformatic tools and/or datasets became available.

### 2.3. Single-Trait Mapping for Behavioral QTLs

The PPI data generated from 455 animals was averaged per BXD strain, which was then used for QTL mapping. Genotypic markers were regressed against each PPI trait using WebQTL. Empirical significance thresholds from 1000 to 1,000,000 permutations of trait data were employed to deal with the genome-wide multiple testing problems inherent in QTL mapping studies [35]. Where significant QTLs were observed, interval mapping was performed to identify the location of the QTL and candidate genes within the QTL region. We then used GEMMA 0.98.5 (genome-wide efficient mixed-model analysis) mapping (http://gn2.genenetwork.org) to confirm the QTL positions. This linear mixed model corrects for overlap in genomes due to kinship (i.e., BXD RI strains are derived from two parental strains and thus highly related genetically) and possible overlaps in gene expression due to interactions between genes and environment [36,37].

### 2.4. Criteria for Identification of Candidate Genes

A 2-LOD confidence interval was used to identify potential candidate genes for PPI. To prioritize candidate genes, we employed a scoring system (scores ranging from 0 to 10) using five different parameters, as follows: (a) significant correlation with PPI (score = 2); (b) containing nonsynonymous SNPs or indels [38] (score = 2); (c) mean expression ≥ 8 in datasets for brain regions associated with schizophrenia and PPI (two-fold higher than the baseline expression level of 7), consistent with methods/strategies we have used previously [39,40] (score = 1); (d) *cis-regulation* (gene located within +/− 10 Mb of flanking SNPs) (score = 2); and (e) functional significance to PPI (score = 3), based on resources including Mouse Genome Informatics (MGI, http://www.informatics.jax.org/) [41], the Rat Genome Database (RGD, www.rgd.mcw.edu) [42], the International Mouse Phenotyping Consortium (IMPC, http://www.mousephenotype.org/) [43], the GWAS Catalog (www.ebi.ac.uk/gwas) [44], and the Kyoto Encyclopedia of Genes and Genomes (KEGG, https://www.genome.jp/kegg/) [45]. The gene with the highest score was selected as the candidate modulating PPI and used for further analysis.

### 2.5. Gene Expression Datasets

Initial analyses used the following publicly available BXD RI expression datasets to examine associations between transcript levels and PPI: prefrontal cortex (PFC) mRNA: VCU BXD PFC Sal M430 2.0 (Dec 06) RMA; hippocampus (HIPP) mRNA: Hippocampus Consortium M430v2 (June06) PDNN; striatum (STR) mRNA: BIDMC/UTHSC Dev Striatum P3 ILMv6.2 (Nov11) RankInv; nucleus accumbens (NAc) mRNA: VCU BXD NAc Sal M430 2.0 (Oct07) RMA; midbrain (Mdb) mRNA: VU BXD Midbrain Agilent SurePrint G3 Mouse GE (May12) Quantile; ventral tegmental area (VTA) mRNA: VCU BXD VTA Sal M430 2.0 (Jun09) RMA; amygdala (AMYG) mRNA: INIA Amygdala Cohort Affy MoGene 1.0ST (Mar11) RMA; and hypothalamus (HYPO) mRNA: INIA Hypothalamus Affy MoGene 1.0ST (Nov10) RMA. Specific information on these datasets (i.e., strain, sex, data processing, etc.) can be found on the GeneNetwork website (www.genenetwork.org). The HIPP, PFC, AMYG, and NAc are implicated in PPI modulation, while the VTA has been implicated in the acoustic startle response, as reviewed in [20].

### 2.6. Functional Analysis

Genetic, partial, and literature correlations were performed to filter lists of transcripts correlated with our candidate gene and to perform gene enrichment analyses [39,40]. To identify genes co-expressed with our candidate gene, we compared the candidate gene’s expression to that of all available probe-sets in the brain regions examined. Genes with expression levels greater than the baseline of 7.0 and correlated with our candidate gene were analyzed further. Following genetic correlation, partial correlation analysis was performed to eliminate any genes that were only genetically, but not biologically, related to the candidate gene. A literature correlation was performed to further examine the correlation between other genes and the candidate based on MEDLINE abstracts and titles [46]. Gene enrichment analysis was conducted using significantly correlated genes with a *p*-value < 0.05, mean expression ≥ 8.0, and literature correlation ≥ 0.2. Gene sets were uploaded to WebGestalt v2024 (https://www.webgestalt.org/, accessed on 10 June 2025), and *p*-values of the over-represented categories were adjusted for multiple comparisons [47]. In addition, we submitted the correlated genes to the Metascape v3.5 (http://metascape.org, accessed on 3 May 2025) [48] to explore networks of Gene Ontology biological processes (GO-BP).

### 2.7. Correlation and PheWAS Analyses

Correlations between PPI and the expression of the candidate gene were examined and Pearson’s product correlation values of *p* < 0.05 were used.

We queried human PheWAS (phenome-wide association study) data for the strongest candidate gene in our QTL region to identify human phenotype associations [49,50], using the PheWAS tool in GWASatlas Release 3 (https://atlas.ctglab.nl/PheWAS, accessed on 15 May 2025) [51].

### 2.8. Protein–Protein Interaction Network Analysis

The genes correlated significantly (*p*-value < 0.05, mean expression ≥ 8, and literature correlation ≥ 0.2) with the candidate gene in the NAc dataset were used to explore interactions at the protein level using the STRING database [52,53], where only “experimentally verified” interactions (medium confidence score of 0.4) were considered. The protein–protein network was further analyzed and visualized using Cytoscape v3.10.1 [54], and network statistics, such as degree, betweenness and closeness centrality, were calculated for each protein in the network.

### 2.9. Validation of Candidate Genes Using Human RNA Sequencing Data

Expression of the candidate genes was validated using human RNA-seq data from schizophrenia patients, retrieved from the Gene Expression Omnibus (GEO) database [55]. Raw read count data of schizophrenia and matched control samples for NAc were downloaded from the dataset GSE202537 (https://www.ncbi.nlm.nih.gov/geo/query/acc.cgi?acc=GSE202537, accessed on 11 November 2024) [56]. Briefly, the postmortem samples were sequenced using Illumina NextSeq 500, raw reads were aligned to the human genome (GRh38) using HISAT2v2.1.0 [57], and read counts were estimated using HTSeq v0.10.0 [58]. The read counts were normalized, and differential expression analysis was performed using DeSeq2 [59]. Genes with an adjusted *p*-value < 0.05 (Benjamini & Hochberg, B&H, correction) were considered significant.

## 3. Results

### 3.1. Prepulse Inhibition

**Figure 1:**
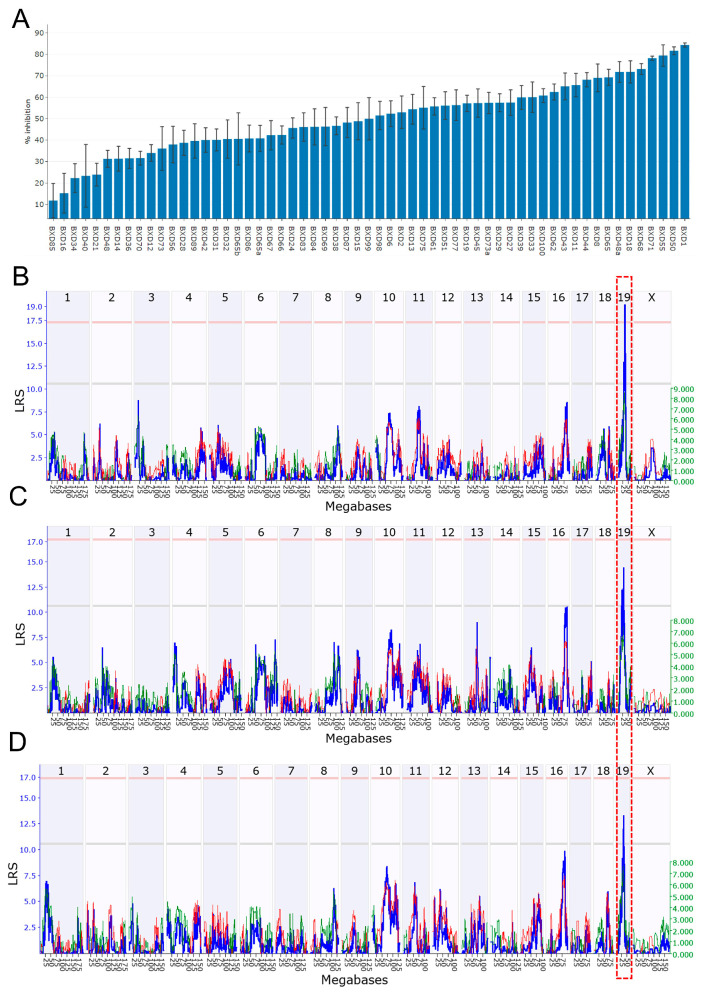
Genetic mapping for prepulse inhibition (PPI) percentage in BXD mice. (**A**) Bar plot showing %PPI in BXD mice at 85 dB. The *x*-axis shows the BXD strains, and the *y*-axis shows %PPI genomic loci mapped across all mouse chromosomes for %PPI at (**B**) 85 dB, (**C**) 70 dB, and (**D**) 80 dB, respectively, in male BXD mice. The *x*-axis indicates the genomic position, while the *y*-axis shows the LRS value, a measurement of the linkage between the % PPI and genomic region. One unit of LRS is equivalent to 4.61 units of “logarithm of the odds” (which is equal to −log10P likelihood ratio). The gray and red horizontal lines indicate suggestive and significant genome-wide thresholds, respectively. Suggestive QTLs were identified for % PPI at 70 and 80 dB, whereas a significant QTL was identified at 85 dB. The overlap of all the three QTLs on Chr 19 is shown with a red dotted box.

The strain distribution for %PPI at 85 dB is presented in Figure 1A; BXD85 had the lowest %PPI (11.78 ± 8.00) and BXD01 had the highest (84.37 ± 0.96). QTL mapping revealed a significant locus for %PPI at 85 dB (GN Trait 11428) between 27 and 36 Mb of Chr 19, with a peak at 32.7 Mb (LRS = 19.2), in male BXD mice (Figure 1B). We also found overlapping suggestive QTLs on the same chromosome for %PPI at 70 dB (GN Trait 11426) and 80 dB (GN Trait 11427) in males. The suggestive locus for GN Trait 11426 was located at 27.53 Mb on Chr 19 (LRS = 14.4) and the locus for GN Trait 11427 at 32.74 Mb (LRS = 13.3) (Figure 1C,D), strongly indicating the association of this chromosomal region with PPI, particularly in male mice.

### 3.2. Identification of Candidate Genes

The 2-LOD region for the QTL of %PPI at 85 dB is located between 27.5 and 36 Mb of Chromosome 19, with ~100 genes in this region. After filtering by genetic correlation with %PPI 85 dB, only ten genes remained (Table 1). Among these, we shortlisted genes with expression levels of at least 8 (reflecting two-fold baseline) in the brain expression datasets, then determined whether genes had functional polymorphisms between B6 and D2 (i.e., non-synonymous, insertions, or deletions in coding or regulatory regions) or a significant *cis*-regulated expression QTL (cis-eQTL). Finally, the functional significance of these ten genes was assessed based on their association with brain/nervous system/schizophrenia-related pathways or phenotypes. Genes were then scored as indicated in the Methods. Of the 10 genes, 8 received more than 50% of the possible total score. *Atad1* (also known as thorase) was the strongest potential candidate gene, with a maximum score of 10. *Rnls* and *Prkg1* received scores of 9 and 8, respectively, while *Asah2* received a score of 7 (Table 1). We found that *Atad1* had expression levels >8 in each of the brain expression datasets examined; however, six of the datasets were not considered further because expression of *Atad1* was not cis-regulated (PFC, HYPO) or expression of Atad1 was not correlated with PPI (STR, MDB, VTA, HYPO)

### 3.3. Genetic, Partial, and Literature Correlations for Identified Candidate and Gene Sets

We queried gene expression dataset [GN156: VCU BXD NAc Sal M430 2.0 (Oct07) RMA] for the top 15,000 genes whose expression correlated with *Atad1*. Partial correlation yielded 7880 genes significantly correlated (*p* < 0.05) with *Atad1* (1148763_at) in the NAc dataset. Next, including those with literature correlations ≥0.20 yielded 2212 genes. Finally, filtering out correlated genes with low expression (≤8) resulted in 1716 genes, which were used for further analysis. We also queried the gene expression dataset [GN 112: Hippocampus Consortium M430v2 (6 June) PDNN], which resulted in a small set of genes, hence our focus on the NAc dataset.

### 3.4. Gene Enrichment Analysis

**Figure 2:**
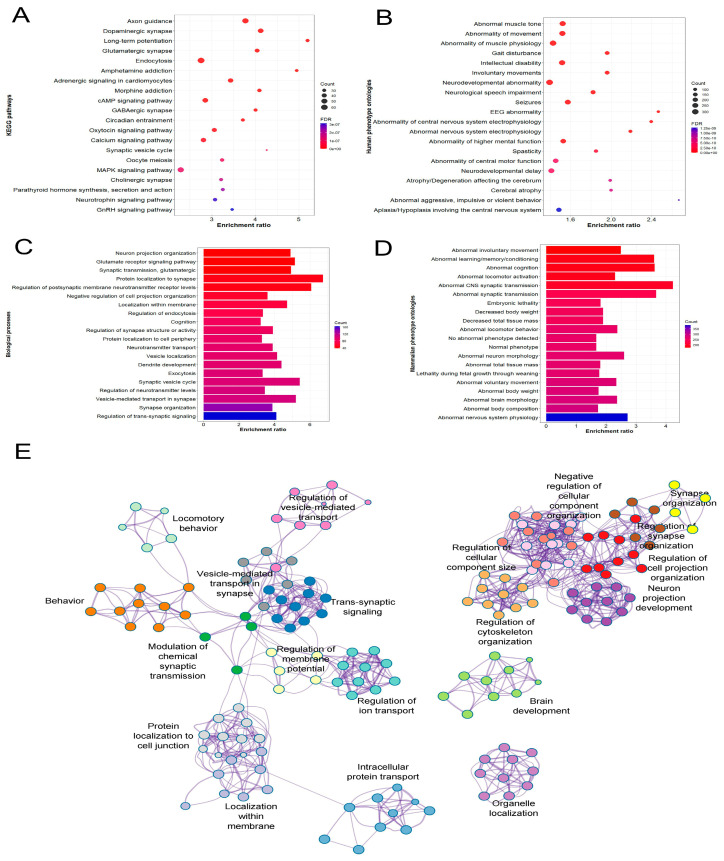
Functional enrichment analysis of Atad1-correlated genes. The genes correlated with Atad1 in NAc dataset were used as input in WebGestalt for enrichment analysis. Top 20 (**A**) KEGG pathways, (**B**) human phenotype ontologies (HPOs), (**C**) Gene Ontology biological processes (GO-BPs), and (**D**) mammalian phenotype ontologies (MPOs), respectively. The top 20 GO-BPs and MPOs presented here are significant with an FDR *p*-value < 0.001. A complete list of functional annotations is provided in Supplementary Table S1. (**E**) The correlated genes were uploaded into the Metscape tool to analyze the network of GO-BPs. Metascape employs a heuristic algorithm to select the most informative terms from the GO clusters obtained. More specifically, it samples the 20 top-score clusters, selects up to 10 of the best-scoring terms (lowest *p*-values) within each cluster, then connects all term pairs with Kappa similarity above 0.3. The nodes represent enriched GO-BP terms and are colored by their cluster IDs. The edges link similar terms. The most significant term of the cluster is displayed as a label to represent that cluster.

Many of the enriched categories for *Atad1*-correlated genes were related to neurotransmission and/or properties of its components. Figure 2A–D show the top 20 significant KEGG pathways, mammalian phenotype ontologies (MPOs), Gene Ontology biological processes (GO-BPs), and human phenotype ontologies (HPOs) enriched by *Atad1*-correlated genes (a complete list of significant annotations, false discovery rate (FDR) *p* < 0.05, is provided in Supplementary Table S1). Among the top KEGG pathways, over 60% were directly associated with nervous system/physiology. “Dopaminergic synapse” and “Axon guidance” were the most significant KEGG pathways, with a *p*-value < 0.001. Similarly, more than 70% of the top 20 GO-BPs were related to neuron/brain-related processes. While the FDR-corrected *p*-value was <0.00001 for the top 20 GO-BPs, “regulation of trans-synaptic signaling” involved the largest number of genes (*n* = 165), followed by “synapse organization” (*n* = 139). Further, our results demonstrated the involvement of *Atad1*-correlated genes in nervous system- and brain-related phenotypes in mice and humans. “Abnormal nervous system physiology” was the most significant MPO, with 383 genes (Figure 2D). The top enriched HPOs were “Involuntary movements”, “Neurological speech impairment”, and “Neurodevelopmental abnormality”. To gain a better insight into the relationships between different biological processes, we constructed a network of GO-BPs. This revealed a close interaction between terms associated with neurodevelopmental physiology. Furthermore, many GO-BP clusters were connected to behavioral processes (Figure 2E).

### 3.5. Phenotype Correlation

**Figure 3:**
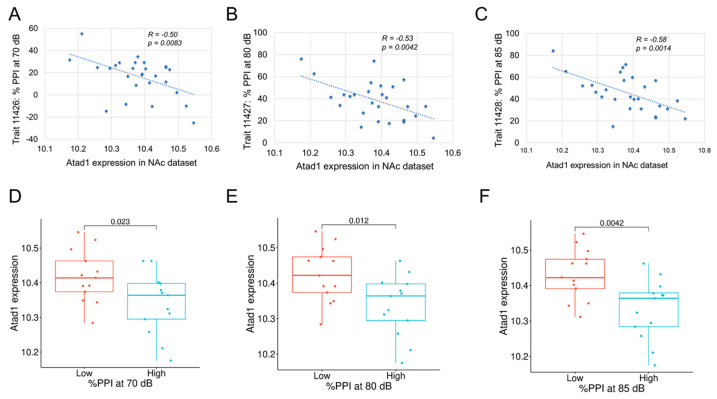
Correlation of prepulse inhibition (PPI) traits in male BXD mice with archived Atad1 expression in nucleus accumbens (NAc). % PPI at (**A**) 70 dB, (**B**) 80 dB, and (**C**) 85 dB is significantly negatively correlated with Atad1 expression. Correlation R and *p*-values are indicated in the figures. (**D**–**F**): *Atad1* expression difference between the “low” and “high” PPI BXD strains. The *p*-value based on Student’s t-test is shown above each plot. More details on traits can be found by querying GeneNetwork with the trait identifiers.

As shown in Figure 3A, the correlation between *Atad1* expression in the NAc and our PPI trait (Trait 11428) was r = −0.582 (*p* = 0.0014), indicating increased expression levels of *Atad1* correlated with lower levels of PPI. Additionally, *Atad1* was significantly negatively correlated with %PPI at 70 dB (GN Trait 11426) and at 80 dB (GN Trait 11427) in BXD mice (Figure 3B,C), strengthening the key role of this gene in PPI. Furthermore, to investigate whether the expression of *Atad1* varied between the BXD strains depending on PPI, we divided the BXD strains into “high” and “low” PPI groups. The grouping was based on the median PPI values across the BXD strains, i.e., strains with PPI values less than the median were classified as belonging to the “low” group, whereas those with PPI values higher than the median were considered as belonging to the “high” group. *Atad1* expression was found to be statistically significant between the two groups based on Student’s *t*-test, and was higher in the “low” PPI group compared to the “high” group for all the traits tested (Figure 3D–F), corroborating with the correlation analysis results.

### 3.6. Protein–Protein Interaction Network

**Figure 4:**
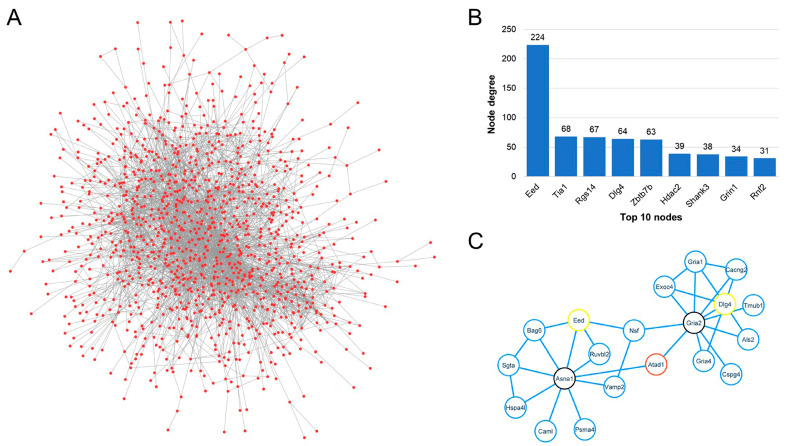
Protein–protein interaction network analysis of Atad1-correlated genes in the NAc dataset. (**A**) The protein–protein interaction network of all Atad1-correlated genes (global network). The network contains 1124 proteins (nodes/dots) and 3507 edges (interactions/lines). The network shows a high number of interactions among Atad1-correlated genes. (**B**) The top 10 nodes based on degree (number of interacting partners). (**C**) Protein–protein interaction network showing primary and secondary interactors of Atad1 (extracted from the global network presented in “panel A”). The Atad1 protein is indicated with a red border; its direct/primary interactors, Asna1 and Gria2, are indicated with black borders; and its secondary interactors, Eed and Dlg4, which have a high node degree in the global network (panel B), are indicated with yellow borders.

We constructed a protein–protein interaction network using *Atad1*-correlated genes to understand how *Ata*d1 affects neurodevelopmental processes. The global network of *Atad1*-correlated genes contained ~1100 proteins and 3500 edges (Figure 4A). A large number of interactions demonstrate that many of these genes work closely together to perform similar functions. This was further corroborated by analyzing network statistics, which revealed that, on average, every protein in the network has a minimum of six interactions (node degree (ND) = 6). EED (embryonic ectoderm development), a protein involved in epigenetic regulation, had the highest ND of 224 (Figure 4B). Other important proteins with high NDs were TIA1 (ND = 68), RGS14 (ND = 67), DLG4 (ND = 64), and ZBTB7B (ND = 63). Our candidate, ATAD1, interacted with only two proteins, but had a “closeness centrality” of 0.25 (maximum value in the global network = 0.44). “Closeness centrality” estimates how fast the flow of information is through a given node to other nodes in a protein interaction network. Although ATAD1 has a low ND, it appears to be an important protein in the network, likely functioning through other molecules. Hence, we explored the ATAD1-specific interaction network by extracting its primary and secondary interactors from the global network. ATAD1 directly interacts with GRIA2 and ASNA1 (Figure 4C). Further, GRIA2 directly interacts with 10 other proteins, including EED, which has the highest ND in the global network. Similarly, ASNA1 interacts with nine other proteins, including DLG4, which has an ND of 64 in the global network. Thus, our protein interaction analysis demonstrated that instead of directly interacting with many proteins, ATAD1 carries out its functions by indirectly interacting with other proteins through GRIA2 and ASNA1. A complete list of interactions and node properties can be found in Supplementary Table S2.

### 3.7. PheWAS Analysis

**Figure 5:**
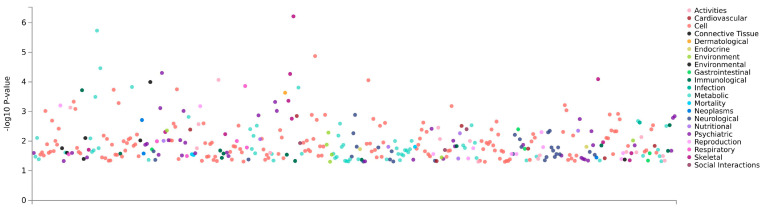
PheWAS analysis for Atad1. This analysis utilizes the Atad1 genomic region to find its associations with phenotypes measured in GWAS datasets. The PheWAS tool within GWASatlas (https://atlas.ctglab.nl/PheWAS, accessed on 15 May 2025) was used for this analysis.

We performed PheWAS analysis (48, 49) using human GWAS datasets. *ATAD1* had significant associations with human phenotypes, including neurological traits (Figure 5). Psychiatric traits significantly associated with *ATAD1* were schizophrenia, bipolar disorder, and depression (Table 2), confirming its association with human neurodevelopmental disorders.

### 3.8. Candidate Variants in Atad1

There are 185 small variants within *Atad1* which segregate in the BXD population (154 SNPs, 15 insertions, and 16 deletions), and evidence of a large 214 bp deletion (32,738,422–32,738,636). None of these variants are in protein coding regions, and the large deletion is within the first intron. Most of the small variants are intronic (*n* = 158); 14 are upstream or downstream of the gene, 12 are in the 3′ UTR, and 1 is in the 5′ UTR. This agrees with our finding that *Atad1* is differentially expressed between the two genotypes (B6 and D2 progenitor strains of the BXD population) and is *cis*-regulated. We are not able to determine which variant is causal, but it is probable that a variant in the 3′ UTR could alter transcription factor binding.

### 3.9. Validation of the Candidate Gene in Schizophrenia Patients

**Figure 6:**
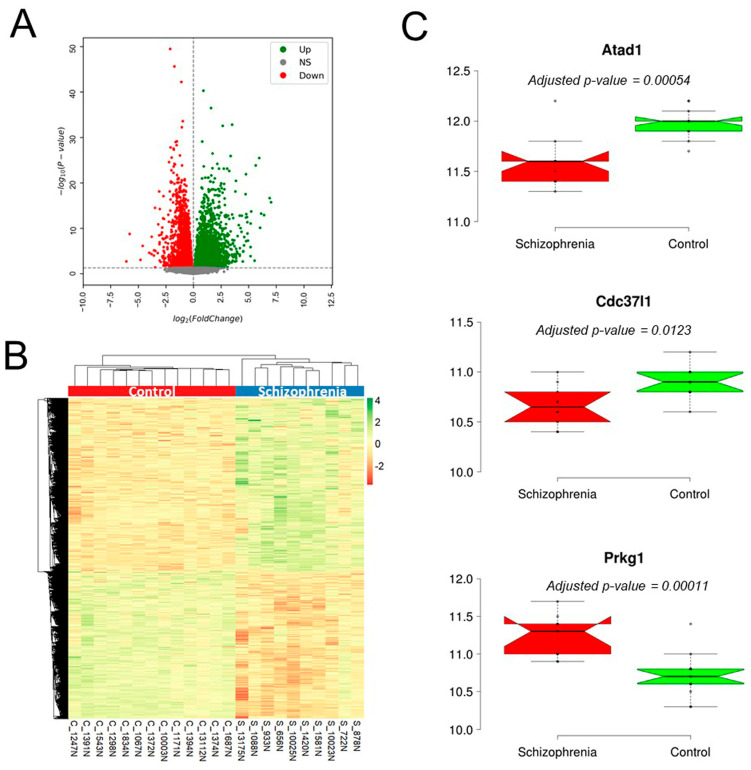
Validation of candidate genes identified in BXD mice using schizophrenia patient RNA-seq data. (**A**,**B**) Volcano plot and heatmap showing significant differential expression of genes between schizophrenia patients and controls. (**C**) Box plots indicating the expression of candidate genes in patient and control groups.

We used the RNA-seq dataset GSE202537 to explore differential expression of candidate genes between schizophrenia (*n* = 28) and control (*n* = 36) NAc samples. We first excluded the outlier samples based on PCA and clustering methods, and finally used 10 schizophrenia and 13 control samples for differential expression analysis. A total of 8132 genes were differentially expressed (4397 upregulated and 3735 downregulated) between the two groups (B&H adjusted, *p* < 0.05) (Figure 6A,B). Of the 10 potential candidate genes (Table 1), 3 genes (*Atad1, Cdc37l1*, and *Prkg1*) were differentially expressed between schizophrenia patients and the control group. While *Atad1* and *Cdc37l1* showed lower expression in the patient than in the control group, *Prkg1* had a contrasting expression pattern (Figure 6C).

## 4. Discussion

Although a significant number of genes have been implicated in schizophrenia and related traits, the etiology of schizophrenia remains poorly understood. Diagnosis and therefore treatment for this disorder is often delayed. Identification of biomarkers may facilitate earlier diagnosis. Because of its robustness, heritability, and ease of study in animal models, PPI is an informative endophenotype of schizophrenia and other psychiatric disorders. Here, we used a powerful genetic resource, the BXD RI panel, to identify genetic factors influencing PPI. The limitations of our use of a single endophenotype are acknowledged, although robust deficits in PPI have been replicated in human sample cohorts [17,60]. Here, we focus not on deficits per se, but on genetic regulation of PPI. We identified a significant male-specific QTL for PPI on Chr 19 in the BXD RI panel and identified *Atad1* as a strong potential candidate gene. Other sex-dependent associations between genes and schizophrenia include *Fabp7*, which is associated with NMDA receptors in mice [27]; in humans, *DLG1*, which codes for a synapse-associated protein, has a male-specific association with schizophrenia [61], and *Ptpn5*, tyrosine-protein phosphatase non-receptor type 5, is important in excitatory postsynaptic activity [62]. These findings suggest the need for closer examination of sex differences in the expression of genes and sex-based variants in genes associated with PPI, schizophrenia, and related traits. Additionally, although we tested relatively young animals and cannot rule out any influence of hearing, potential differences in hearing sensitivity are acknowledged, given that age-related hearing loss has been documented in the BXD parental strains, B6 and D2 [63,64]. However, it should be noted that our PPI QTL did not map to any regions, i.e., Chrs 5, 11, 18 [63,65,66], previously associated with age-related hearing loss in the parental or BXD RI strains. Interestingly, a locus that protects against hearing loss in a small number of strains has been identified on Chr. 16 in the BXD RI strains [64].

Although several schizophrenia susceptibility/risk genes have been identified, researchers have shown that “top” susceptibility genes code for proteins that converge on a highly interconnected molecular network. The heterogeneous nature of schizophrenia, therefore, could be explained by a mutation in any single gene in that highly interconnected network, rather than some common mutation [67] in the large number of genes that have been associated with schizophrenia. The task of understanding how genes function (and especially interact with one another) in such networks remains. The robustness and stability of the PPI phenotype [18,68], coupled with the genetic power of the BXD RI panel, should aid in mapping and elucidating neural and genetic substrates related to sensory gating. Strengthening the argument for the use of PPI as an endophenotype, 117 genetic variants have been associated with sensory/sensorimotor gating, as reviewed in [69]. A critical next step is to discern the mechanisms through which *Atad1* affects PPI. In other words, what is the functional effect? Our protein–protein interaction analysis suggests that *Atad1* functions through its interactions with other highly interconnected players in the network. Gene enrichment analysis revealed that the top enriched category in *Atad1*’s gene network was “dopaminergic synapse”, with “glutamatergic synapse” as the fourth. Reviews have summarized evidence from mutant mice (with gene disruptions) for the putative roles of dopaminergic and glutamatergic pathophysiology in schizophrenia [70,71]. This suggests that Atad1 interacts, indirectly, with other genes that affect neurotransmitter systems, components, and pathways implicated in schizophrenia and other disorders.

In an examination of schizophrenia-related endophenotypes in humans, both distinct and overlapping genes were associated with individual endophenotypes, suggesting that both unique and overlapping pathways are involved in schizophrenia risk [72]. An investigation of the protein interactome found that schizophrenia-related risk genes formed a disease module including genes related to developmental biology and cognition [73]. Postmortem examination of co-expression networks in the schizophrenia prefrontal cortex revealed that genes with altered expression were associated with oxidative phosphorylation, myelination, synaptic transmission, and immune function [74]. The potential PPI QTL gene, *Fabp7*, is associated with functional links to the NMDA receptor [27]; further analysis, using engineered mice and human pluripotent stem cells, revealed a potential role of *Cdh23* in PPI and CDH23 in schizophrenia [75]. The recent identification of *APBB1lP* as a candidate gene for PPI also links immune function with schizophrenia [76]; other studies highlight the role of immune activation and synapse remodeling in schizophrenia [77].

There is mounting evidence that *Atad1* plays a significant role in schizophrenia-related traits. The construction of glutamatergic synapses throughout development is critical in the etiology of schizophrenia [78]. *Atad1* is associated with glutamatergic signaling and regulation of AMPA receptor (AMPAR) expression. AMPAR trafficking is implicated in synaptic plasticity/learning and memory [78], and may be key in understanding cognitive function and dysfunction [79]. More recent studies have highlighted the importance of *ATAD1* in the regulation of neurodevelopment and synaptic function [80]. In a mouse model, loss of ATAD1 increased surface expression of AMPARs, which was directly related to increased AMPA current. While its loss did not affect basal transmission at CA1 synapses, long-term potentiation (LTP) increased and long-term depression (LTD) was blocked. Further, aberrant behaviors in the open field and Y-maze suggest that *Atad1*-KO mice (deletion limited to forebrain structures) showed short-term memory deficits specific to novel environments [81]. Other studies suggest that rapid GluR1 (an AMPAR subunit) trafficking may be required for short-term memory processes [82]. *Atad1* likely prevents the recycling of GluR1 (and GluR2) back to the plasma membrane [76]. Recent studies have identified *ATAD1* variants in the postmortem brains of schizophrenia patients. These findings were followed up with mouse studies that showed that AMPAR-mediated synaptic transmission was increased after deletion of ATAD1 proteins from dopamine neurons. Moreover, the conditional loss of this gene (cKO) impaired LTD at glutamatergic synapses onto dopamine neurons, showing that glutamatergic transmission onto dopamine neurons is important to fear learning, but not generalized fear [83]; the cKO mice also showed greater associative learning and enhanced second-order conditioning in comparison to wildtype littermates. Likewise, associative learning deficits (contextual and cued fear conditioning) were observed in heterozygous mice expressing *Atad1* variants; these mice displayed deficits in spatial working memory, spatial recognition memory, and PPI, and social behavior deficits were reversed by perampanel, an AMPAR antagonist [84]. Such findings are important because *Atad1* variants can affect the disassembly of the AMPAR/Glutamate interacting protein-1 (GRIP1) complex [84], which is important in AMPAR trafficking, particularly internalization of AMPARs. We identified several variants in *Atad1*. A limitation is that we are not able to pinpoint the causal variant(s) in *Atad1*; however, others have shown that genetic variants in untranslated mRNA regions can modify regulatory mechanisms (i.e., transcription, structure, localization) and contribute to disease processes [85]. Thus, an important next step is determining which of the identified *Atad1* variants influence PPI.

## 5. Conclusions

We identified *Atad*1 as a candidate gene for PPI and demonstrated that *Atad1*-correlated genes are enriched in annotations related to behavior, synapse function, and brain development. Human mapping studies have not implicated *ATAD1* in schizophrenia and related disorders; however, this gene has been implicated in neuronal function, synaptic organization, and transmission, among others [6]. Recent mouse studies, however, have shown that ATAD1 signaling is important in preventing α-synucleinopathy and behaviors associated with Parkinson’s Disease [86]. We identified associations between *ATAD1* and psychiatric traits in human GWAS datasets. Finally, using an RNA-seq dataset, we found differential expression of *ATAD1,* including two other candidates, *CDC37L1 *and *PRKG1*, between schizophrenia and control groups, suggesting that examination of co-regulation of *Atad1* and other “players” is warranted. Future studies should further characterize the role of *Atad1* in PPI, schizophrenia, and related phenotypes, i.e., *ATAD1* has been associated with hyperekplexia, a phenotype associated with “exaggerated” startle and excessive stiffness [87,88,89,90,91].

## Figures and Tables

**Table 1:** Candidate genes within the QTL region and significantly correlated with % PPI at 85 dB.

Official Symbol	Mean Expression ≥ 8 (Score = 1)	Non-Syn SNPs/Indels (Score = 2)	Cis-Regulation (Score = 2)	Sig. Phenotype Correlation (Score = 2)	Functional Significance (Score = 3)	Total (Score = 10)
Atad1	Yes	Yes	Yes	Yes	Yes	**10**
Rnls	--	Yes	Yes	Yes	Yes	**9**
Prkg1	Yes	Yes	--	Yes	Yes	**8**
Asah2	--	Yes	--	Yes	Yes	**7**
Ifit1	--	Yes	--	Yes	Yes	**7**
Cdc3711	Yes	--	--	Yes	Yes	**6**
Ak3	Yes	--	--	Yes	Yes	**6**
Papps2	Yes	--	Yes	Yes	--	**5**
Ifit1bl2	--	--	--	Yes	Yes	**5**
Ranbp6	Yes	--	--	Yes	--	**3**

**Table 2:** Psychiatric traits significantly associated with Atad1 based on human GWASs.

Atlas ID	PMID	Year	Trait	*p*-Value	N
12	24280982	2014	Schizophrenia/bipolar disorder	0.040	39,202
15	28439101	2017	Post-traumatic stress disorder	0.013	9223
30	21173776	2012	Agreeableness (NEO-FFI)	0.047	17,375
1141	27494321	2016	Chronotype	0.001	128,266
1142	27494321	2016	Sleep duration	0.045	128,266
1173	26955885	2016	Chronotype (continuous)	4.98 × 10^−5^	100,420
1174	26955885	2016	Extreme chronotype	0.032	100,420
2018	24369049	2014	Lithium response in Bipolar I patients—Alda Scale of 7 to 8	0.042	294
2025	22952603	2012	10 mg response to amphetamine	0.025	381
2043	27329760	2016	Bipolar disorder	0.036	34,950
3230	31427789	2019	Morning/evening person (chronotype)	0.040	345,148
3235	31427789	2019	Current tobacco smoking	0.009	386,150
3262	31427789	2019	Average weekly red wine intake	0.025	274,058
3287	31427789	2019	Sensitivity/hurt feelings	0.042	375,272
3292	31427789	2019	Worry too long after embarrassment	0.002	370,660
3295	31427789	2019	Guilty feelings	0.000	376,361
3296	31427789	2019	Risk-taking	0.031	372,651
3297	31427789	2019	Frequency of depressed mood in last 2 weeks	0.001	370,017
3394	31427789	2019	Ever unenthusiastic/disinterested for a whole week	0.024	123,848
3567	31427789	2019	Why stopped smoking: illness or ill health	0.021	94,509
3655	31427789	2019	Smoking status: previous vs. current	0.014	177,025
3729	31427789	2019	Depression—age at first episode of depression	0.009	65,776
3744	31427789	2019	Cannabis use—ever taken cannabis	0.012	126,632
3770	31427789	2019	Depression—trouble falling or staying asleep or sleeping too much	0.001	126,545
3772	31427789	2019	Depression—recent feelings of tiredness or low energy	0.030	126,540
3775	31427789	2019	Traumatic events—able to pay rent/mortgage as an adult	0.014	124,944
3795	29942085	2018	Neuroticism	0.015	390,278
3796	29942085	2018	Depressive symptoms	0.011	381,455
3982	29483656	2018	Schizophrenia	0.002	105,318
3993	29500382	2018	Irritability (IRR)	0.048	260,369
3999	29500382	2018	Worry too long after embarrassment (WORR-EMB)	0.001	261,094
4002	29500382	2018	Guilty feelings (GUILT)	0.001	265,139
4014	29700475	2018	Major depressive disorder	0.004	173,005
4040	29906448	2018	Schizophrenia/bipolar disorder	0.037	107,620
4087	29255261	2018	Neuroticism	0.010	329,821
4294	30696823	2019	Chronotype	0.029	449,732
4316	30643251	2019	Smoking cessation	0.005	312,821
4368	30150663	2018	Cannabis use	0.013	162,082

## Data Availability

The publicly archived dataset for PPI is available on GeneNetwork, https://genenetwork.org/. The PPI traits analyzed in this paper are GN Trait 11428, GN Trait 11427, and GN 11426. The gene expression datasets examined include the following: GN135: prefrontal cortex mRNA: VCU BXD PFC Sal M430 2.0 (Dec 06) RMA; GN112: hippocampus mRNA: Hippocampus Consortium M430v2 (June06) PDNN; GN377: striatum mRNA: BIDMC/UTHSC Dev Striatum P3 ILMv6.2 (Nov11) RankInv; GN156: VCU BXD NAc Sal M430 2.0 (Oct07) RMA; GN381: midbrain mRNA: VCU BXD Midbrain Agilent SurePrint G3 Mouse GE (May12) Quantile; GN228: ventral tegmental area mRNA: VCU BXD VTA Sal M430 2.0 (Jun09) RMA; GN323: amygdala mRNA: INIA Amygdala Cohort Affy MoGene 1.0ST (Mar11) RMA; and GN281: hypothalamus mRNA: INIA Hypothalamus Affy MoGene 1.0ST (Nov10) RMA. These are also publicly available on GeneNetwork. The RNA-seq dataset is GSE202537.

## Supplementary Materials

The following supporting information can be downloaded at https://www.mdpi.com/article/10.3390/genes16101139/s1. Table S1: Functional annotation of KEGG pathways, HPOs, GO-BPs, and MPOs. Table S2. Protein–protein interactions and node properties.

## Abbreviations

The following abbreviations are used in this manuscript:

PPI	Prepulse Inhibition
QTL	Quantitative Trait Locus
BXD RI	B6 × D2 Recombinant Inbred Strain
B6	C57/BL6
D2	DBA2/J
PheWAS	Phenome-Wide Association Study
GN	GeneNetwork
LRS	Likelihood Ratio Statistic
*Atad1*	ATPase family, AAA domain containing 1, also known as thorase
PFC	Prefrontal Cortex
HIPP	Hippocampus
STR	Striatum
NAc	Nucleus Accumbens
MDB	Midbrain
VTA	Ventral Tegmental Area
AMYG	Amygdala
HYPO	Hypothalamus
GRIA2	Glutamate Ionotropic Receptor AMPA type 2 subunit
ASNA1	Arsenical Pump-Driving ATPase 1
GWAS	Genome-Wide Association Study
*Disc1*	Disrupted in Schizophrenia 1
*Nrg1*	Neuregulin 1
Chr	Chromosome
SNP	Single-Nucleotide Polymorphism
GEMMA	Genome-Wide Efficient Mixed-Model Analysis
LOD	Logarithm Odds
MGI	Mouse Genome Informatics
RGD	Rat Genome Database
IMPC	International Mouse Phenotyping Consortium
KEGG	Kyoto Encyclopedia of Genes and Genomes
mRNA	Messenger RNA
RMA	Robust Multi-Array Average
STRING	Search Tool for the Retrieval of Interacting Genes/Proteins
GEO	Gene Expression Omnibus
*Prkg1*	Protein Kinase cGMP-dependent type 1
*Rnls*	Renalase, FAD Dependent Amine Oxidase
*Asah2*	N-acylsphingosine amidohydrolase 2
*Papss2*	3′-phosphoadenosine 5′ phosphosulfate synthase 2
*Ifit1*	Interferon-induced protein with tetratricopeptide repeats 1
*Cdc37l1*	Cell Division cycle 37 homolog-like 1
*Ak3*	Adenylate Kinase 3
*Ranbp6*	RAN binding protein 6
*Ifit1bl2*	Interferon-induced protein with tetratricopeptide repeats 1B like 2
MPO	Mammalian Phenotype Ontology
GO-BP	Gene Ontology Biological Processes
HPO	Human Phenotype Ontology
FDR	False Discovery Rate
ND	Node Degree
EED	Embryonic Ectoderm Development
TIA1	Cytotoxic Granule-Associated RNA binding protein 1
RGS14	Regulator of G protein Signaling 14
DLG4	Discs Large Homolog 4
ZBTB7B	Zinc finger and BTB domain containing 7B
GRIA2	Glutamate Ionotropic Receptor AMPA type subunit 2
bp	Base pair
UTR	Untranslated Region
PCA	Principal Component Analysis
*Fabp7*	Fatty acid binding protein 7
*Ptpn5*	Tyrosine-Protein-Phosphatase Non-receptor Type 5
APBB1lP	Amyloid Beta Precursor Protein Binding Family B Member 1 Interacting Protein
AMPAR	AMPA receptor
LTD	Long-Term Depression
cKO	Conditional Knockout
GRIP1	AMPAR/Glutamate Interacting-Protein 1
